# A Novel Method for Detection and Progress Assessment of Visual Distortion Caused by Macular Disorder: A Central Serous Chorioretinopathy (CSR) Case Study

**DOI:** 10.3390/vision3040068

**Published:** 2019-12-11

**Authors:** Navid Mohaghegh, Sebastian Magierowski, Ebrahim Ghafar-Zadeh

**Affiliations:** Biologically Inspired Sensors and Actuators (BioSA) Laboratory, Department of EECS, York University, Toronto, ON M3J1P3, Canada; navid@navid.ca (N.M.); magiero@eecs.yorku.ca (S.M.)

**Keywords:** macular disorders, central serous retinopathy (CSR), age-related macular degeneration (AMD), scalable vector graphics (SVG), graphical macular interface system (GTMIS)

## Abstract

This paper presents a new mathematical model along with a measurement platform for accurate detection and monitoring of various visual distortions (VD) caused by macular disorders such as central serous chorioretinopathy (CSR) and age-related macular degeneration (AMD). This platform projects a series of graphical patterns on the patient’s retina and calculates the severity of VDs accordingly. The accuracy of this technique relies on the accurate detection of distorted lines by the patient. We also propose a simple mathematical model to evaluate the VD created by CSR. The model is used as a control for the test results achieved from the proposed platform. The proposed platform consists of the required hardware and software for the generation and projection of patterns along with the collection and processing of patients against their standard optical coherence tomography (OCT) images. Based on these results, the OCT images agree with the VD test results, and the proposed platform can be used as an alternative home monitoring method for various macular disorders.

## 1. Introduction

**Figure 1:**
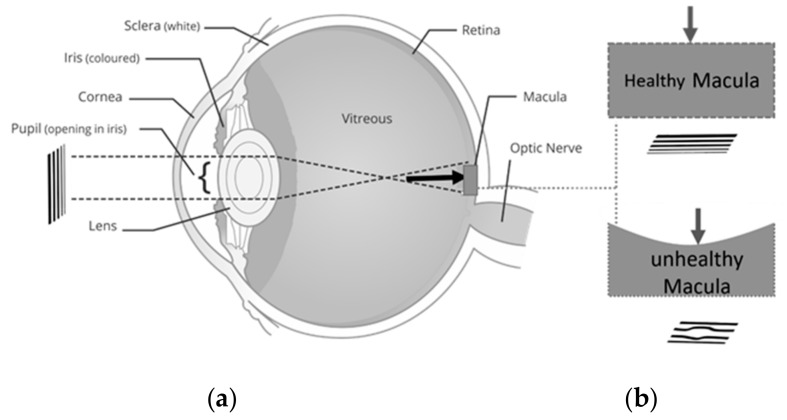
Central serous retinopathy (CSR) development (**a**) eye including sclera, iris, corona, pupil, lens, entire retina, macula, optic nerve, (**b**) created image in normal and CSR affected the retina.

Macular disorders such as myopic maculopathy, age-related macular degeneration (AMD) and central serous retinopathy (CSR) affect central vision [1]. A hundred million people are suffering from different macular disorders including AMD which is the leading cause of blindness for people with macular disorders [1]. In their early stages, these diseases cause visual distortion (VD) as seen in Figure 1. In their advanced stages, these disorders may result in blindness. The VD can be in the form of loss of acuity, loss of contrast sensitivity or even metamorphopsia (seeing straight horizontal or vertical lines as wavy) and scotoma (partial loss of vision in some areas of the visual field). The VD in any form has significant impacts in the everyday life of a patient. Therefore, the detection of VD in the early stages of development is crucial for eye care and treatment. In this paper, we propose a low complexity, home monitoring VD detection, and progress assessment method. The focus of this work is placed on CSR as a case study; however, in the future, we plan to extend the applicability of the proposed platform for other macular disorders such as AMD. 

*CSR* is a macular condition [2], that is associated with high levels of stress [2,3]. For diagnosis purposes, optical coherence tomography (OCT) and the Amsler Grid chart are used as gold standard diagnostic methods [4]. These methods are employed to assess the visual performance of patient suffering from CSR in order to perceive any new developed VDs. However, this method suffers from low sensitivity in detecting small VDs (50% failure on VDs smaller than six degrees [5]). Additionally, the exact location of VD cannot be identified with using OCT images. On the other hand, the progress of VD cannot directly be accurately quantified using the Amsler chart. 

Computerized graphical VD assessment methods have recently emerged to overcome the above-mentioned problems [6,7,8,9,10]. Despite great advances, there are many challenges in using computerized VD assessment techniques. We discuss these challenges and compare the proposed solutions in the literature in the next section. In this paper, we address the challenges of a high accuracy VD assessment method based on the macular disorder’s VD model. As aforementioned, we use CSR as a case study macular disorder model. 

An easy to use, home monitoring, online VD detection, and progress assessment method not only can help patients to promptly follow-up with their doctors if any significant change is observed in their VD, but also can be used as a platform to collect data from patients suffering from various macular disorders. The collected data may include the VD results plus other questions related to the type of medications used by patients, their diet and other activities during the day. If the experiment can create such a rich source of data for clinical research, pharmaceutical, and other applications will greatly benefit. For instance, the main factors in CSR can be studied on a large number of patients using the proposed online method. To date, many papers have reported the importance of medical big data using available medical information in hospitals, but the challenge of developing new medical big data [11,12,13] using real-time medical measurements remain unmet. This is because of the cost and technical challenges of developing medical devices for continuous, accurate, and easy to use medical sensing purposes. The long-term objective of this work is the development of a novel medical big data center that can collect the VD assessment results and securely store the data in a cloud server. This paper paves the way for the development of new macular big data using proposed VD assessment software and an advanced hardware platform. 

In the remainder of this paper, the related literature is discussed in Section 2. Then, a simplified model of the CSR disorder is proposed in Section 3. This model is used to generate patterns as discussed in Section 4 that describes the details of software and hardware developments required to perform the VD assessment and collect data. The results will be discussed in Section 4, followed up with a conclusion in Section 5.

## 2. Related Works

**Figure 2:**
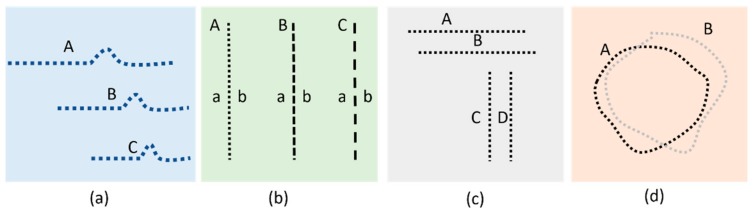
Illustration of stimulating patterns used in (**a**) PHP, (**b**) M-Chart, (**c**) MCPT, and (**d**) CTAG.

Computerized VD methods have recently attracted the attention for an accurate measurement of distortion in macular disorders. These diseases may result in two different damages in the visual field which are called metamorphopsia and scotoma. Metamorphopsia refers to wavy or blurry VDs. Scotoma refers to missing parts in the visual field (very sever VDs) [14]. To date, many different VD assessment methods such as accelerated Amsler grid (AAG) [15], preferential hyperacuity perimeter (PHP) [7,16,17], Macular Computerized Psychophysical Test (MCPT) [7], and M-CHART [18,19] have addressed the challenge of developing graphical interface techniques to accurately detect defective visual field areas and/or assess their progress [7,20]. Assuming the defective area has already been identified by the ophthalmologist’s assistant manually using the Amsler grid, then in each one of the above techniques, a graphical object is subsequently repeatedly projected in different spots of the affected areas of the visual field. The patient should confirm the perception after each frame verbally or by using control devices such as a keyboard or mouse. Figure 2 illustrates the graphical patterns that are shown in each one of the mentioned methods. 

As seen in Figure 2c, for each MCPT frame, a horizontal or vertical straight line is projected upon the retina. Subsequent to a rapid fixation, the patients are asked if they detect any VD in the lines projected to the retinal lesion. Similarly, PHP generates stimuli consisting of a straight line of dots with a very small area of the line out of alignment (Figure 2a). Any VD detected in the displayed lines should be identified subsequently by touching the screen using a stylus pen [7,21]. In the M-CHART method (Figure 2b), vertical dotted lines are shown in different frames with differently sized spaces between the dots (ranging from fine to coarse dotted lines). The patient is asked to confirm if any misalignment is seen for the lines displayed. In all the aforementioned techniques, a series of lines are displayed (in case of PHP, lines containing an artificial bump). 3D-CTAG [22,23] is another technique that can be used to detect the metamorphopsia and scotomas as seen in Figure 2d. A patient is asked to mark the VD area on the Amsler grid. The process is repeated for different contrast levels of the grid. 

The above-mentioned VD assessment methods are simple, non-invasive, and suitable for home diagnostic purposes. Some limitations associated with these techniques are as follows. The fixation of patient at the center of the screen can get disturbed. This disturbance of fixation diminishes the accuracy of VD detection. The required time of examination will also be increased. It is noteworthy to mention that any pointing task has an inherited difficulty that is explained by Fitts’ Law [24] and thus it is preferred to minimize pointing tasks as much as possible. In this paper, we have addressed the aforementioned issues by proposing a new platform to generate and project various patterns and record the verbal responses to avoid the change of fixation and eliminate pointing tasks. It is noteworthy that the accuracy of aforementioned methods, as well as the proposed method in this paper is affected by the fixation stability that is a crucial issue in patients with neovascular age-related macular degeneration [25]. 

## 3. CSR VD Changes 

The physical changes in the retina due to macular disorders can significantly affect visual performance. The deformation of the retina due to macular disorders exert forces in cellular layers. These forces can displace the cells related to each point of the visual field. Therefore the straight lines might be seen curvy [3,4,20]. On the other hand, deformation of the retina might slightly deviate it from the focal point of the lens. This may change sharpness, contrast, and brightness of patients’ vision. Due to the complexity of the visual effects of these disorders, it is not possible to precisely quantify the effect of VDs and consequently predict the observed images by the patient. In this section, we propose a low complexity model of VD in the visual field of the patient suffering from CSR by taking into account one of the abovementioned effects—curvy lines. Given the fact that the accuracy of the computerized VD graphical methods relies on the accurate detection of distorted lines, it is crucial to have an idea of the distorted images seen by the patient. This model can be utilized to design better patterns for VD tests. 

### 3.1. Effect of CSR in the Visual Field

**Figure 3:**
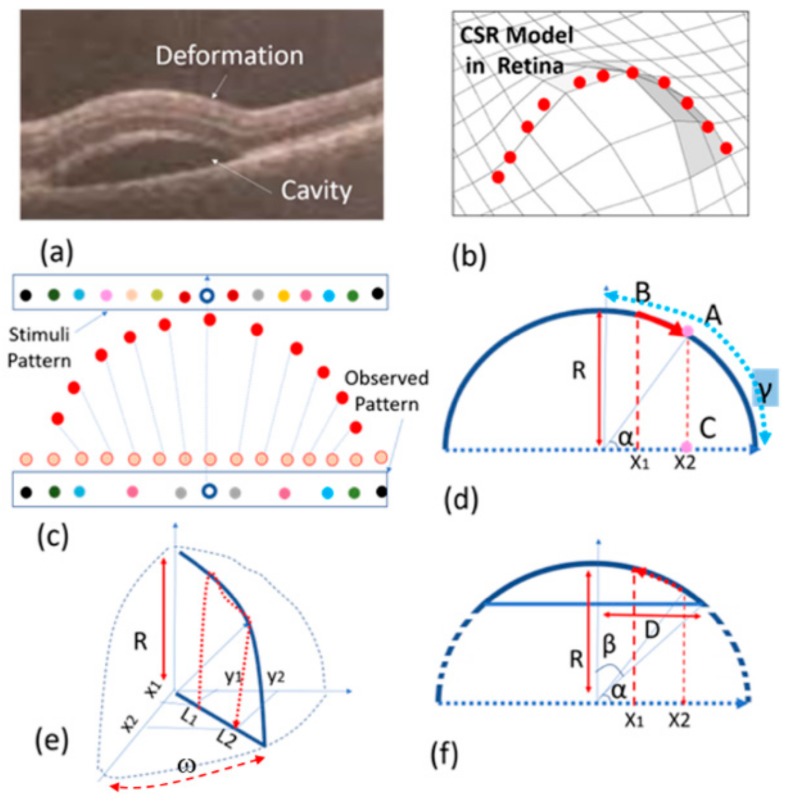
Proposed model: (**a**) OCT image of a CSR cavity, (**b**) a semi-spherical model, (**c**,**d**) half-spherical shape activity using discrete and continuous presentation, (**e**) three-dimensional (3D) half-spherical shape activity with partially spherical shape cavity model, (**f**) semi-spherical–cylindrical shape CSR cavity mode.

The creation of CSR is due to the collection of fluid under the basement of the retina. This results in the creation of micrometer-scale structures such as a cavity as seen in Figure 3a. This figure shows OCT images of the retina affected by CSR. The structure can be modeled with spherical or semi-spherical geometries. One may argue that the deformation of the retina can affect the cones, rods, and other components between the photoreceptors and the basement of the retina including the entire retinal pigment epithelium layers. As demonstrated in Figure 3b, the deformation of the retina due to CSR can slightly stretch the retina layers and consequently displace the visual sensing points on the top layer. These small displacements can cause significant VD. In the next subsection, we derive the mathematical relationships for modelling a half-spherical shape’s deformation. Even though the CSR structure does not exactly assume this shape, our simple model can be extended to estimate the VD caused by CSR. 

A CSR deformation can be modelled with a sphere as illustrated in Figure 3b–d. Naturally, the cross-section of this deformation assumes a half-circular locus. Given the fact that the number of sensing points or ‘pixels’ in the retina is the same before (orange points in Figure 3c) and after deformation (red points in Figure 3c), we can assume these sensing points are distributed uniformly before and after retinal stretching. For this reason, as a result of this deformation, the observed pattern in the retina differs from the pattern stimulated or observed in the normal retina. In other words, the distance between the sensing points in the deformed retina will be different from the one in the normal retina. Therefore, the stimuli pattern will be different from the pattern observed by the patient (see Figure 3c). In other words, the light from each point of the stimuli pattern that reaches the deflected retina at point x_1_ is where the light will be observed by the patient in point x_2_ (see Figure 3d). 

### 3.2. Spherical Shape CSR Modeling

With reference to Figure 3d, *x_1_* can be obtained from the following equation.

(1) $$\frac{x_{1}}{R}=\frac{\alpha R}{\left(\gamma \right)R}$$

where *R* is the sphere’s radius, γ = π/2 and α are the angles between the sensing point and horizontal axis that become equal to $\pi x_{2}/2R$ using Equation (1). On the other hand, x_2_ is equal to $R\sin\left(\alpha \right)$, thus x_2_ becomes equal to $R\sin\left(\pi x_{2}/2R\right)$ that is a one-dimensional model of displacement of the visual point from x_1_ to x_2_. However, the real visual point should be modelled in two dimensions as seen in Figure 3e. In this figure *L_1_* and *L_2_* replace x_1_ and x_2_ so that $L_{1}=\sqrt{x_{1}^{2}+y_{1}^{2}}$ and $L_{2}=\sqrt{x_{2}^{2}+y_{2}^{2}}$ where (x_1_, y_1_) are the cartesian coordinates of the object point seen in a normal retina. This point is displaced to (x_2_, y_2_) so that $L_{1}=R\sin\left(\pi L_{2}/2R\right)$. On the other hand, tan(ω) = y_1_/x_1_ = y_2_/x_2_ where ω is an angle shown in Figure 3e. By combining the above information, Equations (2) and (3) are obtained.

(2) $$x_{2}-x_{0}=\frac{2\left(x_{1}-x_{0}\right)R}{\pi \sqrt{{\left(x_{1}-x_{0}\right)}^{2}+{\left(y_{1}-y_{0}\right)}^{2}}\;}.\sin(\frac{\gamma \sqrt{{\left(x_{1}-x_{0}\right)}^{2}+{\left(y_{1}-y_{0}\right)}^{2}}}{2R})$$

(3) $$y_{2}-y_{0}=\frac{2\left(x_{1}-x_{0}\right)R}{\pi \sqrt{{\left(x_{1}-x_{0}\right)}^{2}+{\left(y_{1}-y_{0}\right)}^{2}}\;}.\sin(\frac{\gamma \sqrt{{\left(x_{1}-x_{0}\right)}^{2}+{\left(y_{1}-y_{0}\right)}^{2}}}{2R})$$

where $\left(x_{0},\;y_{0}\right)$ is the center of spherical VD area. Equations (2) and (3) are used to obtain the displacement of the stimulation pattern where ${\left(x_{1}-x_{0}\right)}^{2}+{\left(y_{1}-y_{0}\right)}^{2}$ < R^2^. The actual CSR deformation shape as seen in OCT image in Figure 3a, can be modelled with a semi-sphere shape structure. With reference to x_1_ in Figure 3e–f, γ = β = sin^−1^(D/2R), therefore Equations (2) and (3) with this having γ can be re-written to obtain the displacement of (x_1_, y_1_) to (x_2_, y_2_). 

In the aforementioned spherical shape CSR modelling, we discussed a simple model that has been extracted from the OCT images of human subjects in order to create the required patterns for the detection of macular disorders. As will be described in the next section, we use this modelling technique to estimate the distortion of a pattern seen by the patient. This can help to design patterns with maximum observable distortion by patients. 

## 4. Proposed Method and Platform 

**Figure 4:**
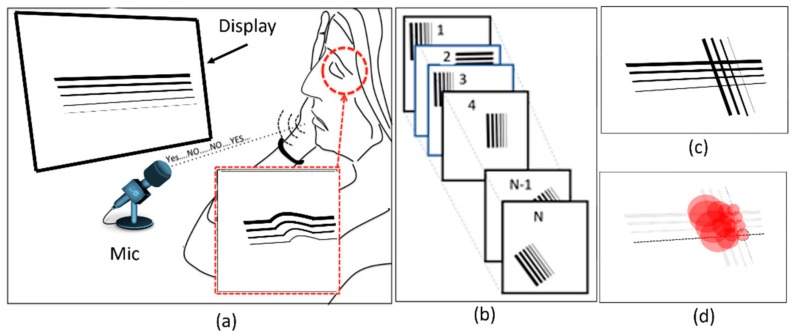
Proposed method: (**a**) Projecting a pattern in each frame and recording the response of the patient from (**b**) a series of frames, and (**c**,**d**) creating a heatmap showing the maximum possibility of VD.

This section presents the proposed method for the detection of VD in the visual field using a unified software and hardware platform named NGRID. The proposed platform relies on displaying a series of frames with predefined patterns (Figure 4a). Each frame is represented with a matrix F_m×n_(i) with *m* column and *n* rows and index *i* ranging from 1 to N that refers to the number of test-pattern frames presented to the patient (Figure 4b). The patient response is collected as illustrated in Figure 4a. The test patterns are designed in a way that presents itself as distorted to patients suffering from CSR. OCT images of the patient along with the proposed VD models are utilized to create the VD test patterns. By displaying each pattern, the patient can say ‘good’ or ‘bad’ if the pattern is seen as normal or distorted, respectively. A series of responses associated with the displayed patterns are recorded using a voice recognition system (Figure 4a). 

This platform is used to recognize and convert the responses to a series of 1 s and 0 s (‘good’ and ‘bad’ response is converted to binary data ‘1’ and ‘0’, respectively). These responses (Figure 4c) are used to generate a graph, namely a ‘heatmap’, as seen in Figure 4d. This heatmap is used to evaluate the progress of VD in the retina. The purpose of the generated heatmap is to highlight the damaged regions in the retina. In this work, we developed the platform’s hardware and software to perform the VD test. 

### 4.1. Hardware Development 

**Figure 5:**
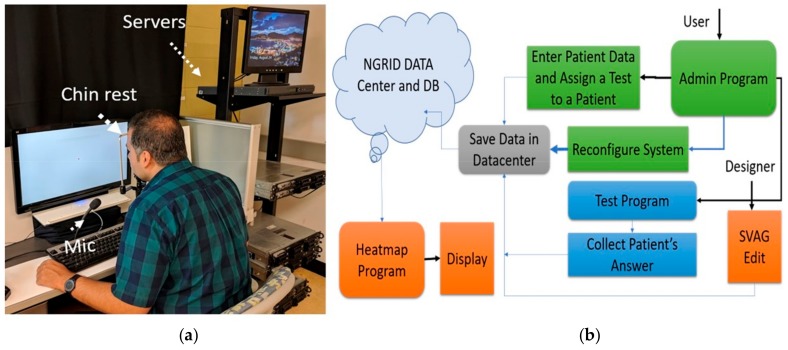
The proposed platform with (**a**) hardware setup and (**b**) software process flow.

The hardware includes a server cluster, voice recognition system, display monitors. Figure 5a shows this setup. A highly available (HA) cluster of servers was set up to host our application. Each server consists of 64 GB of RAM and two physical Intel Xeon 5460 CPUs along with four Gbps host bus adapter (HBA) card to access our storage area network (SAN). We used Linux containers (LXC) and Linux virtual server (LVS) to create an HA cluster that we can run our server-side application including admin interface, database, and codes related to heatmap generator programs. The details of the proposed hardware in this work was reported in [26]. 

### 4.2. Software Development 

The primary function of the proposed software is presented in Figure 5b. This software allows users access to the platform and the ability to run the VD tests or to administrate aspects of the VD test program. These programs, referred to as admin programs, were developed to manage and provide services to three groups of users including clinical users (patient, nurse or ophthalmologist), the engineering designer, and main system administrator. The data and test results are securely stored in the cloud infrastructure (provided by the hardware server cluster). The administrator interface allows adding new patients to the system, as well as assigning them different VD tests. The admin interface also allows the creation of various VD graphical tests through SVG VD test editor. The results are post-processed by the heatmap generator and analyzer programs which also run on the cloud infrastructure. Table 1 lists the programs included in the software platform with a brief description of programming languages and tools that are used. 

#### 4.2.1. NGRID VD Test and SVG VD Test Editor

NGRID VD test is a program that projects tests to the patient retina and collects the answers. This program renders the VD vector patterns created by the SVG VD test editor for the patient in order to do the VD tests. A series of N frames created in the SVG VD test editor is displayed on the screen and a detailed log of voice responses is also collected and converted to a series of 0 s and 1 s to be post-processed for heatmap analysis. 

**Figure 6:**
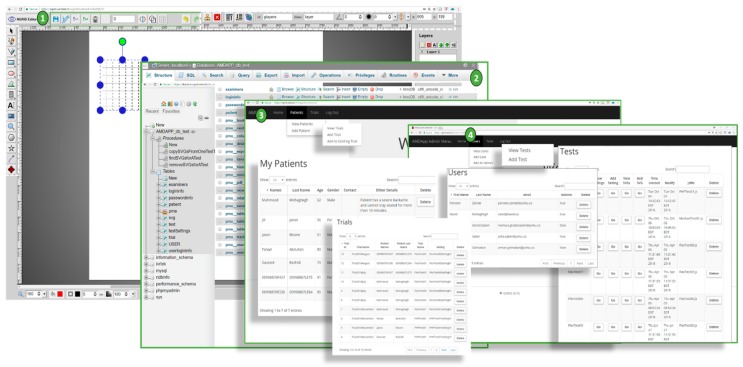
The developed programs: (**1**)—Customized SVG editor, (**2**)—Database access interface and admin user interfaces (**3**,**4**).

The SVG VD test editor is the editor that is used to create different test graphical patterns that compose a VD test. Each pattern includes a number of straight lines. A pattern editor allows generating a vector-based drawing of the straight-lines and editing the style of the lines to create a VD test frame. This graphical editor exposes the Cartesian coordinates of the points used to create the patterns in each frame and allows an easy way of editing the overall vector graphics. We used scalable vector graphics (SVG) [27] as the main standard for the underlying graphics. SVG allows the generation of patterns that are independent of different display screens used in rendering the VD test. This allows the test design to be done more easily and to be decoupled from aspects such as canvas size, resolution, and pixel density of the rendering VD test displays. In this work, we customized the SVG editor that allows for easy and rapid creation, as well as saving the tests online. An important advantage of this editor is the online accessibility and flexibility in selecting the width, colour, angle, and the overall style of the patterns. Figure 6(1) shows the customized SVG VD test editor used for the creation of patterns. Using this editor, a series of N frames are generated and saved as a VD test. 

#### 4.2.2. Admin Interface

A customized online administrative interface is designed to enter the required data such as patient information, assigned trials, as well as authorization access to SVG VD test editor and database of VD test repositories (Figure 6(2)). As shown in Figure 6(3), the user interface is designed to allow the patients, ophthalmology assistants, and system engineers to enter patient and VD test data. They can also control the access levels and update various configuration levels as shown in Figure 6(4) (e.g., selection and changes to the VD test patterns, managing the style, colors, and width of each line, duration time (t) of a VD test, as well as the rate (r) of the VD test during projection on retina via head-mounted displays). Admin interface programs were written in Scala with a smart caching layer to save the most frequently used queries or VD tests to allow more efficient access to test data.

### 4.3. Heatmap Generator

In the first step of generating the heatmap, all frames associated with ‘bad’ responses are collected and collapsed in a single matrix called *C_mxn_*. Let us assume a series of N bits is represented by a matrix R_N_ including responses (*R_j_* = 0 or 1) of the patient to the visualized N frames *F_mxn_(i)* where 1 ≤ i ≤ N can be obtained from the following equation.

(4) $$C_{m\times n}=\sum_{i=1}^{i=N}R_{i}\cdot F_{mxn}\left(i\right)$$

This matrix shows all locations of damages in the retina. The value of each member/pixel of C shows the number of distorted patterns meeting in the pixel. C matrix is used to generate the heatmap and includes a circle in each pixel. Each pixel of the C matrix is proportional to the radius and opacity of that circle. A complex program was developed to generate the heatmap along with normalization processes to better distinguish between the damage and normal areas in the heatmap graph (we used the z-scores while subtracting each pixel’s value from the mean-value matrix, clamping the negative z-score values, and dividing the value of each pixel with the maximum value in the z-score matrix). 

### 4.4. Other Programs 

A wide variety of other programs were written to enable the NGRID platform. Significantly, we highlight the voice recording and recognition layer that allows the rapid detection of a limited lexicon of words in a soft real-time fashion. This is crucial to the overall platform as we mainly use the patient’s voice to interact with the VD test program, as well as projecting the next pattern upon receiving the patient’s response. We developed a native MS Windows application for this purpose to not only facilitate speech recognition and voice recording (for audit purposes), but also integrate with the eye-tracker (to assure the patient is looking at the center of the screen). This program also acted as a soft real-time event-hub for various time-critical inputs and facilitates logging of events with millisecond accuracy. The log data is securely uploaded to our cloud server cluster for heatmap post-processing and analysis. We discuss the heatmap results in the next section. 

## 5. Implementation and VD Test Results

In this section, we demonstrate and discuss the simulation results related to CSR models. The modeling program along with the pattern generator editor was used to generate the patterns that are demonstrated and briefly discussed in this section. Additionally, we put forward the details of VD tests using human participants. 

### 5.1. Simulation Results of CSR Modeling

**Figure 7:**
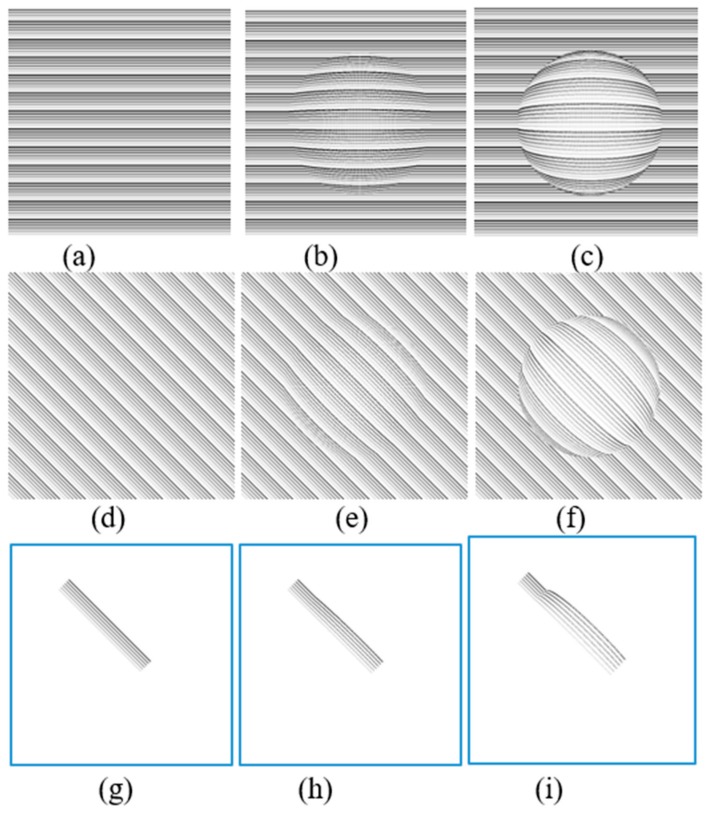
Simulation results of semi-spherical (**b**,**e**,**h**) and spherical (**c**,**f**,**i**) models on three different patterns (**a**,**d**,**g**).

The effect of CSR models including half-spherical and semi-spherical models on three predefined patterns are shown in Figure 7. In these simulations, R = 250 and D = 60 and 125 pixels for modeling purposes. By assuming the patterns (Figure 7a,d,g) are projected upon the retina, Figure 7b,f,h estimate the distorted images seen by a patient suffering from mild CSR (D = 60). On the other hand, Figure 7c,g,i estimate that the distorted images are seen by a patient suffering from severe CSR using (D = 125). 

The simulation VD patterns in Figure 7g are similar to the ones shown in each frame to patients in our proposed method described in Section 4 (Figure 4b). The estimated distortions in the spherical shape model are shown in Figure 7h,i. Based on these results, the straight lines are distorted similarly to a CSR patient that may experience VDs. This is why a group of straight lines can be used to detect the VDs in the visual field. Additionally based on the simulation results shown in Figure 7, the severity of the deformation of the VDs experienced in the test lines are dependent on how large the CSR cavities are (D = 60 vs. D = 125). As can be seen in Figure 7h, in early stages the distortion can hardly be observed.

**Figure 8:**
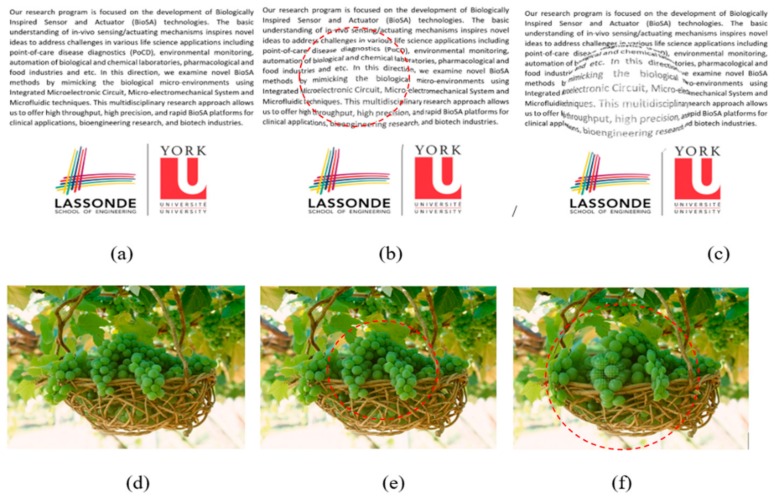
VD simulation results of semi-spherical (**b**,**e**) and spherical (**c**,**f**) models on three different patterns (**a**,**d**). The dot line circles show the distorted areas in (**b**,**e**,**f**).

Figure 8 aims to display the same simulation on more natural scenery that patients experience in their day to day life. Figure 8a is presenting a sample text while Figure 8d is showing a sample photo that is seen by the normal eye. As seen in Figure 8b,e, the distortions are lower (D = 60) than the distortions seen in Figure 8c,f (D = 125). Another interesting point is that the distortion can be better recognized in the text (Figure 8b,c) than in the photo (Figure 8e,f). Overall the simulation results are shown in Figure 7, and Figure 8 can confirm the advantage of simple patterns such as straight lines or text to be projected in the patient’s retina for the VD tests.

### 5.2. Visual Distortion Test

In this work, we performed the VDT1 test on a patient suffering from CSR. This test is composed of a total of 167 frames. The heatmap was generated using the patient responses; these results are demonstrated, discussed, and compared with OCT images with the same patient. It is noteworthy that OCT is a non-invasive medical imaging technique. This low-coherence interferometry employs a near-infrared light to create optical cross-sections of the retina. Given the fact that these images are high resolution and allow changes in retinal thickness to be seen, OCT can play an important role in evaluating CSR and other macular disorders. The subject is a 48-year-old male suffering from CSR. The rapid change of his macular condition allowed us to repeat the VD tests and compare with OCT follow up for observation of changes. 

**Figure 9:**
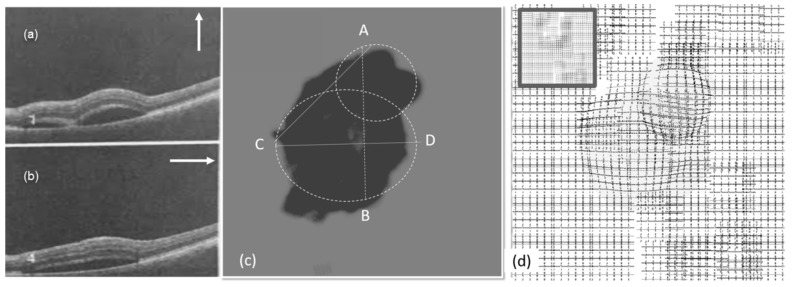
Experimental results (left eye, first visit): (**a**,**b**) OCT images, (**c**) heatmap graph, and (**d**) estimated VD using the CSR model.

**Figure 10:**
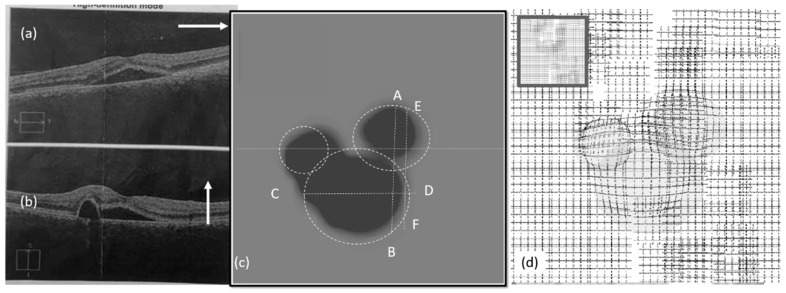
Experimental results (left eye, second visit): (**a**,**b**) OCT images, (**c**) heatmap graph, and (**d**) estimated VD using the CSR model.

**Figure 11:**
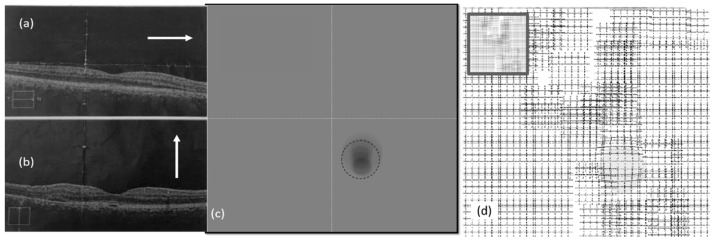
Experimental results (right eye, second visit): (**a**,**b**) OCT images, (**c**) heatmap graph, and (**d**) estimated VD using the CSR model.

The subject performed the VD test, and his responses were collected, post-processed, and heatmaps were generated using the heatmap generator program. The subject visited the Sunnybrook hospital emergency room (ER) while observing a large yellow circle in the center of his vision. The subject also complained about metamorphopsia upon seeing the paper-based Amsler grid. AMD and CSR were the initial diagnoses based on the symptoms. However, OCT images from his left and right eye revealed a major and minor CSR macular condition, respectively. After the first and second visit, the VD test (VDT1) was performed, and the corresponding heatmap graphs were compared with OCT images as seen in Figure 9, Figure 10 and Figure 11.

Figure 9a,b show the OCT images of the patient from the left eye in the first visit. The OCT images were taken in the vertical and horizontal cross section in the directions shown in Figure 9a,b. Figure 9c shows the heatmap graph, based on the number of ‘bad’ and ‘good’ responses described in the last section. As seen in the heatmap, two CSR lesions can be modeled. Based on the results shown in Figure 9, the VD test patterns can be customized for the patient so that each pattern is placed around the estimated area. This can reduce the required time of the test by decreasing the number of frames. 

Figure 9d shows the effect of the CSR on groups of test patterns used in VDT1 based on the generated heatmap. These results estimate the VD of the patient suffering from CSR. The accuracy of this process varies depending on the accurate observation and detection of deformation by patients. 

Figure 10a,b show the OCT images of the left eye in the second visit. The VD test was performed and the corresponding heatmap graph shown in Figure 10c. The effect of the CSR model is modeled in Figure 10d. Figure 11 shows the same for the right eye of the patient.

As seen in Figure 9, Figure 10 and Figure 11, the vertical (AB) and horizontal (CD) cross section of OCT images agree with the corresponding OCT images. Based on the results in Figure 10c, by changing the vertical cross-section from AB, to EF, likely only one cavity related to the distorted upper area could be seen. Similarly, the direction of AC in Figure 9c is in agreement with OCT images displayed in Figure 9a. 

As seen in OCT images for the second visit (Figure 11b), there are minimal changes to the retina of the right eye due to CSR. This means the patient may not experience metamorphopsia in the right eye. This is why the patient did not complain about his right eye in the first or even second visits. However, NGRID VDT1 detected some VDs as seen in Figure 11c. This VD detection via NGRID test can prove the importance of the proposed method for detecting the VDs even when the patient does not experience severe metamorphopsia. 

### 5.3. Estimation of Visual Distortion

**Figure 12:**
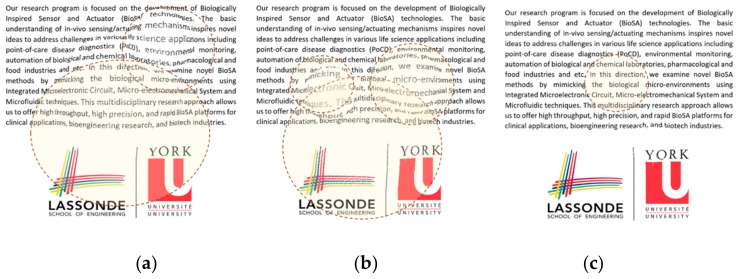
Observed images by the patients suffering from CSR. In the (**a**) first visit left eye, (**b**) in the second visit left eye, and (**c**) in the second visit right eye.

Similar to the results shown in Figure 9d, Figure 10d, and Figure 11d, we can estimate the VD in the left and/or right eye in the two visits. We applied the same model on a picture as seen in Figure 12a–c. These images show the variation of the observed image by the patients in different tests. As seen in these figures, the VD is changing from one test to another due to the change of the CSR cavity and the fluid build-up between the upper retina layers and its basement. Interestingly, we find it very difficult for a normal eye with the corrected vision to notice any VDs in Figure 12c while NGRID could detect the presence of this small VD. 

It is noteworthy to mention that the observed distortion modeled in each of the above Figure 9d, Figure 10d, and Figure 11d along with their equivalent Figure 12a–c is calculated using the total area covered by the heat circles (Figure 9c, Figure 10c, and Figure 11c) to the total area of the heatmap. The percentages are calculated using the heatmap program which counts the number of heat pixels (N) in each one heatmap has seen in Figure 9c, Figure 10c, and Figure 11c and divides per the total number of pixels that is equal to M = 800 × 800 pixels in this paper. Therefore, the distortion (η) on each heatmap is equal to 100 × N/M. Based on this relaxation η is about 27%, 24%, and 2% in the heatmap observed in Figure 9c, Figure 10c, and Figure 11c, respectively. Similarly, the distortion observed in Figure 12a–c approximately are 27%, 24%, and 2%, respectively. 

## 6. Conclusions

In this paper, we proposed a computerized VD assessment method for monitoring the presence and progress of macular disorders including CSR. We put forward a unified software/hardware platform for performing the VD test. We also described the proposed platform consisting of the projection of patterns, secure collection of patient responses using voice recognition, converting the voice response to data for estimating the VD by generating the heatmap. In this work, an SVG editor was also developed for generating the patterns that are placed individually on a number of frames. We also proposed a novel CSR model for estimating the VD. This model can help to more accurately generate the patterns using the SVG editor. We demonstrated and discussed the simulation and experimental results. With successful preliminary experimental results, we have paved the way for running a clinical trial on a large number of patients in the next 12–18 months. The ethical research board in Sunnybrook hospital has approved the clinical trial. The new clinical tests are critical for the optimization of the proposed method. Based on the simulation results, we successfully presented how a CSR disorder can cause VD and distort the straight lines. On the other hand, the experimental results including the CSR OCT images and corresponding heatmaps of the left and right eye of an unhealthy subject. Based on these results the OCT images are in agreement with heatmap graphs. These results also proved that the heatmap could be used to estimate the distortion region in the visual field and accordingly the observed patterns, as well as other images seen by the patient suffering from CSR. The proposed software, hardware, and model in this paper offer an easy to use, and home monitoring method for the detection and progress assessment of macular disorder. 

## Figures and Tables

**Table 1:** A summary of programs developed in the software platform.

Program	Language	Input/Process/Output
VD Test	C++ and C#	Consumes stored VD tests patterns from online admin interface and produces raw test results stored in the cloud data center cluster.
SVG VD Test Editor	HTML5, CSS3 and Javascript	Facilitates creating various SVGs that compose patterns used in tests. The outputs are stored in database
Admin Interface	Scala, Java, HTML5/CSS3 and Javascript	An online website that allows physicians and admins to login and manage various patients and control a different aspect of the VD test.
Heatmap Generator and Analyzer	R, Python, C#, C++, OpenCV, HTML5, CSS3 and Javascript	Consumes raw VD test results along with various rasterization and image processing techniques to produce heatmap results for a test that a patient has completed.
Database	SQL and NoSQL	A highly available database within datacenter that keeps all test data, hashed credentials and test results.
